# Plasma Lipidomic Profiles Improve upon Traditional Risk Factors for the Prediction of Arterial Stiffness Among Patients with Type 2 Diabetes Mellitum: A Randomized, Placebo-Controlled Trial

**DOI:** 10.3390/nu16213618

**Published:** 2024-10-25

**Authors:** Jiaju He, Zhongying Li, Rui Li, Xiaowei Ma, Xiaomin Sun

**Affiliations:** 1 College of Information Science and Technology, Beijing University of Chemical Technology, Beijing 100029, China; 2021040052@mail.buct.edu.cn; 2 Global Health Institute, Xi’an Jiaotong University Health Science Center, Xi’an 710061, China; lzying1215@163.com (Z.L.); lruismile@163.com (R.L.); 3 School of Public Health, Xi’an Jiaotong University Health Science Center, Xi’an 710061, China; mxw_xjtu_edu@163.com

**Keywords:** exercise, vitamin D, diabetes, arterial stiffness, lipidomics

## Abstract

Background: Exercise or vitamin D intervention can reduce the risk of arterial stiffness; however, the underlying mechanisms of lipid metabolism remain unexplored. To examine the effects of a 12-week moderate and vigorous exercise program (65–80% maximal heart rate, 60 min/time, 2~3 times/week) with or without vitamin D supplementation (1000 IU/day) on the reduction in arterial stiffness and further explore whether the effects of interventions could be associated with the basal lipidome among patients with Type 2 diabetes mellitum (T2DM). Method: 61 patients with T2DM were randomly assigned to the following groups: control (CON, *n* = 15), exercise (EX, *n* = 14), vitamin D (VD, *n* = 16), and exercise + vitamin D (EX + VD, *n* = 16). Arterial stiffness risk factors (ankle–brachial index (ABI); brachial–ankle pulse wave velocity (baPWV), systolic blood pressure (SBP), and diastolic blood pressure (DBP)) were evaluated before and after the intervention. The plasma lipidome was determined using ultra-performance liquid chromatography coupled with tandem mass spectrometry. Machine learning was applied to establish prediction models for the responsiveness to arterial stiffness. Result: Vitamin D supplementation could inhibit the decrease in the ankle–brachial index (mean ± SD: EX + VD and VD, −0.001 ± 0.058; EX + CON, −0.047 ± −0.089; *p* = 0.03). We observed high inter-individual variability in the arterial stiffness risk factors in response to the interventions. We also found that optimally selecting the lipid predictors at baseline, such as SM d44:6, LPE 18:2, and Hex2Cer 29:0, could enhance the predictive power by 100% for arm SBP changes in the exercise group. Basal levels of Cer (33:1) and GM3 (44:4) could enhance the predictive power by 100% for changes in baPWV in the vitamin D group. Conclusions: A 12-week vitamin D supplementation was beneficial in preventing arterial stiffness. Compared with traditional clinical risk factors, specific lipids at baseline could significantly improve the ability to predict intervention-induced changes in the reduction of arterial stiffness.

## 1. Introduction

The prevalence of diabetes has increased worldwide. In China, the prevalence of diabetes has dramatically increased from 2.6% in 2002 to 9.7% in 2010 and 11.9% in 2018 [1], imposing a heavy burden on China’s healthcare system [2]. Arterial stiffness (AS) is an independent risk factor for cardiovascular diseases (CVDs) and mortality [3,4]. Individuals with prediabetes and diabetes have a higher risk of AS than those with normal blood glucose levels [5]. When diabetes is accompanied by AS, the risks of CVDs and mortality increase further [6].

Vitamin D supplementation plays a role in reducing the risk of AS and CVDs, regardless of its regulatory effects on calcium homeostasis and mineral metabolism [7,8]. Clinical trials and animal experiments have reported that vitamin D supplementation can decrease blood pressure (BP) by inhibiting the renin–angiotensin–aldosterone system’s activity, modulating vascular wall function, and reducing vascular oxidative stress [9]. Observational studies have revealed a significant correlation between serum 25(OH)D concentrations and two other primary indicators of atherosclerosis: pulse wave velocity (PWV) and the augmentation index (AI). Some interventional studies have also demonstrated the positive effects of vitamin D supplementation on PWV and/or AI [10,11,12,13].

Recent studies have reported that exercise could increase serum 25(OH)D concentrations and the expression of muscular vitamin D receptors [14,15,16]. Although both endurance and resistance exercise can improve the renin–angiotensin–aldosterone system’s activity, moderate endurance exercise is considered as the first choice for patients with T2DM; the American College of Sports Medicine and the American Diabetes Association’s guidelines also state that endurance training with moderate intensity is an essential strategy for treatment of T2DM [17]. This evidence suggests that vitamin D supplementation combined with endurance exercise may have a synergistic effect on the reduction in AS [18].

Metabolomics approaches have been used to elucidate the pathophysiology of hypertension and AS [19]. Substantial evidence supports the idea that AS is closely associated with lipid metabolism (e.g., sphingomyelin (SM), ceramides, acylglycerol) [20]. Compared with healthy patients, those with AS exhibit lower levels of certain long-chain phosphatidylcholines and hemolytic phosphatidylcholines, which are negatively correlated with carotid–femoral PWV and heart rate [21]. A study showed that the severity of AS was inversely associated with phosphatidylethanolamine, phosphatidylcholine, phosphatidylserine, and sphingolipid metabolites [22]. An increasing number of studies have suggested that vitamin D or exercise alone plays a non-negligible role in reducing the risk of AS by affecting lipid molecular species [23]. Moreover, our previous study identified that several lipid species alone or in combination with clinical indices largely improved the prediction of changes in glycemic control [24]. The evidence suggests that lipidomic profiling may comprehensively bridge the gaps between vitamin D or exercise interventions and AS risk.

Therefore, we examined the effects of a 12-week endurance exercise training program, with or without vitamin D supplementation, on the reduction in arterial stiffness (AS). Additionally, we investigated whether basal plasma lipidome levels could serve as predictors of individual responses to these interventions, specifically in terms of the reduction in AS.

## 2. Materials and Methods

### 2.1. Trial Design and Participants

This study was originally designed as a 12-week 2 × 2 factorial randomized, placebo-controlled clinical trial (RCT) regarding patients with Type 2 diabetes melitum (T2DM) in Xi’an, China (34° N latitude). The primary aim of our study was to assess the impact of a combined vitamin D and exercise intervention on insulin resistance. Sixty-one patients with T2DM aged 33–65 years who were not on insulin treatment were enrolled from 2017 to 2018. They were randomly allocated into four groups using a computer-generated random number sequence: the exercise + vitamin D group (EX + VD: *n* = 16), the exercise group (EX: *n* = 14), the vitamin D group (VD: *n* = 16), and the placebo control group (CON: *n* = 15).

T2DM was diagnosed on the basis of the 1999 World Health Organization criteria for diabetes [25]. Patients were included in the study if they met the following criteria: (1) a T2DM diagnosis within the past 10 years, without the use of insulin therapy; (2) no consistent vitamin D or calcium supplementation over the last year; (3) absence of regular exercise habits in the preceding year; (4) no history of renal insufficiency, osteoporosis, or fractures; and (5) a lack of significant sun exposure. Exclusion criteria included patients who had acute infections, diabetes-related complications, or metal implants that could interfere with magnetic resonance imaging (MRI) and dual-energy X-ray absorptiometry (DXA) assessments. Patients who planned to alter their medications during the intervention were also excluded. The details have been reported elsewhere [26].

To maintain the integrity of randomization, the trial designers, testers, and data collectors were blinded to the vitamin D or placebo assignments until after the intervention and data collection were finalized. Participants were instructed to avoid engaging in any structured exercise or altering their usual physical activity and dietary habits throughout the intervention period. They were also asked to refrain from consuming caffeine, alcohol, and tobacco, as well as from performing strenuous physical activity prior to blood sample collection. All assessments were conducted at both the baseline and conclusion of the intervention.

**Figure 1:**
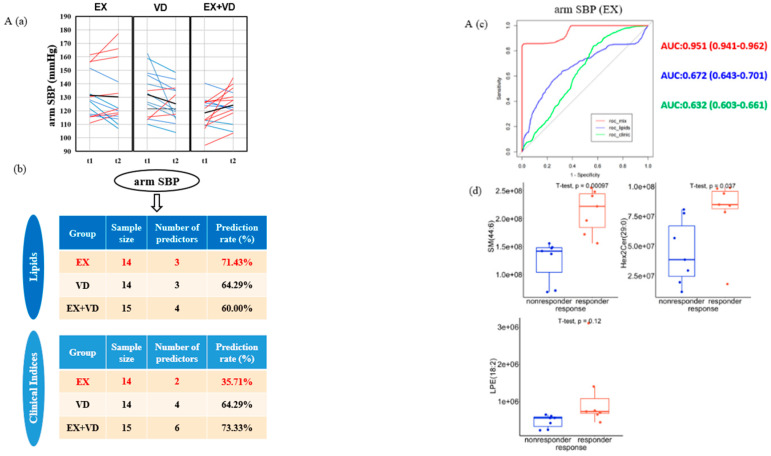

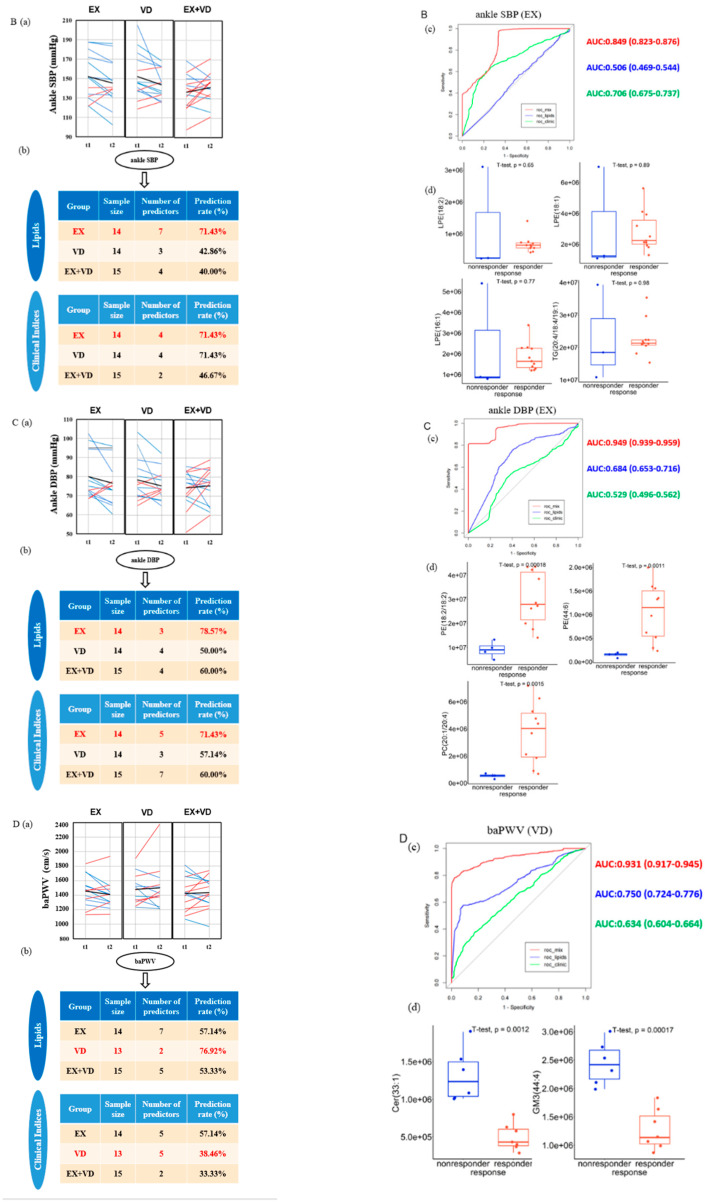
Responders and non-responders and baseline predictions for changes in arterial stiffness ((**A**) arm SBP; (**B**) ankle SBP; (**C**) ankle DBP; (**D**) baPWV). (**a**) Individual variation in changes in arterial stiffness risk across the EX, VD, and EX + VD groups is depicted, with blue lines representing individuals with decreased values, red lines for those with increased values, and black lines indicating no changes. The bold black lines show the change in group means. (**b**) A random forest algorithm, integrated into a repeated double cross-validation framework with unbiased variable selection, was used to predict baseline outcomes for EX and VD. Optimal marker panels were identified for predicting arm SBP. (**c**) Predictive accuracy was evaluated through the misclassification rate (%) and the area under the receiver operating characteristic curve (AROC, 95% confidence interval). (**d**) Baseline levels of predictors of the reduction in arterial stiffness in the EX or VD groups showed differences between responders and non-responders. A Wilcoxon test was applied, with *p* < 0.05 considered statistically significant.

The purpose, procedures, and risks of the study were explained to each participant before inclusion, and written informed consent was obtained. All procedures were reviewed and approved by the Ethics Committee of Xi’an Jiaotong University Health Science Center. The study was conducted in accordance with the Declaration of Helsinki and was registered in the Chinese Clinical Trial System (No. ChiCTR1800015383, registered on 28 March 2018).The complete process is illustrated in Figure 1A. We used the CONSORT reporting guidelines [27].

### 2.2. Interventions

#### 2.2.1. Vitamin D Intervention

Patients in the VD or EX + VD groups received one tablet of a vitamin D3 supplement (1000 IU/day, Nature Made, Otsuka Pharmaceutical Co., Ltd., Tokyo, Japan), and patients in the EX or CON group received a placebo with an identical appearance, shape, and color to the supplement every day. The placebo tablets contained only starch, cellulose, and magnesium stearate. All supplements were prepared in the same bottles and sent to the homes of the participants or were hand-delivered monthly. The participants were instructed to take the tablets after meals. Moreover, they were asked to make a record after taking the supplements daily.

#### 2.2.2. Exercise Intervention

Patients in the EX or EX + VD groups participated in 1 h of progressively increasing aerobic exercise (cycling or rowing) at 65–80% of the maximal heart rate (HRmax) two to three times weekly for 12 weeks. HRmax was calculated using the following formula: HRmax = (220 − age) [28]. Throughout the exercise sessions, the participants’ heart rates were tracked using polar monitors, alongside their rating of perceived exertion (RPE), and adherence to each protocol was documented. The sessions were overseen by a certified trainer who was familiar with the study’s guidelines and procedures. Each session started with a 5 min warm-up on the treadmill, performed at 50–60% of their maximum heart rate (HRmax). This was followed by a 50 min endurance workout, primarily involving cycling, and ended with a 5 min cool-down phase at 40–50% of HRmax, incorporating both cycling and stretching activities. Specifically, the exercise schedules involved 65–70% of HRmax for Week 1, 70–75% of HRmax for Weeks 2–4, 75–80% of HRmax for Weeks 5–8, and 80% of HRmax for Weeks 9–12 (Appendix A, Table A1). The participants in the VD and control groups were asked to maintain their lifestyle habits during the trial. Additional details have been provided in a previous study [29].

#### 2.2.3. Data Collection

##### Anthropometric Measurements and Questionnaires

Height and body mass were measured with the patient barefoot and wearing light clothing. The body mass index (BMI) was calculated as body mass in kilograms divided by height in meters squared (kg/m^2^). Dual energy X-ray absorptiometry (DXA) was used to measure body fat percentage (BF%) (Hologic QDR-4500, DXA Scanner, Hologic Inc., Bedford, MA, USA) with the aid of an experienced operator.

The duration and intensity of the activities of the participants were investigated using the International Physical Activity Questionnaire. The weekly level of a particular physical activity was calculated as the Metabolic Equivalent (MET) assignment corresponding to a single physical activity × weekly frequency (d/w) × daily time (min/d) [30]. Patients were asked to log the duration of their outdoor activity and the areas of skin exposed between 9 a.m. and 5 p.m. over seven consecutive days, both before and after the intervention, using a questionnaire. A score was then calculated to estimate the average weekly sunlight exposure. Further details of this method have been published previously [31]. Demographic data and disease histories were collected using structured questionnaires.

##### Risk Factors of AS (Blood Pressure, Ankle–Brachial Index, and Brachial–Ankle Pulse Wave Velocity)

The systolic blood pressure (SBP), diastolic blood pressure (DBP), ankle–brachial index (ABI), and brachial–ankle pulse wave velocity (baPWV) were measured using a non-invasive vascular screening device (BP-203RPE II device; Omron Healthcare, Kyoto, Japan). The participants were asked to lie flat on the detector and take a five-minute break. The limbs of the participants were tied to appropriate BP cuff sensors and electrocardiograph electrodes, and a phonocardiogram sensor was connected. Each participant was assessed thrice, and the average values of the right and left BP, ABI, and baPWV were automatically calculated and used.

##### Plasma Lipidomic Profiling

Venous blood samples collected before and after the interventions and after the 12-week follow-up were transferred to separate evacuated plastic tubes, centrifuged at 3000 rpm for 10 min at 4 °C, and frozen at −80 °C as plasma aliquots prior to lipidomic analyses. Prior to extraction and instrumental analysis, the order of the plasma samples was randomized to minimize the effects of signal fluctuations. More details regarding the instrumental analysis and data processing procedures have been discussed previously [24].

##### Statistical Analysis

All statistical analyses were conducted using R 4.0.5 or SPSS 24.0.

Descriptive statistics were calculated using the mean (standard deviation) for continuous variables and *n* (%) for categorical variables within a 95% confidence interval (CI). Baseline characteristics among groups were compared using one-way analysis of variance for continuous variables and chi-squared tests for categorical variables. The paired Student’s t-test was used to compare AS risk factors within groups. A 2 × 2 factorial design was used to estimate the effects of vitamin D supplementation and the exercise intervention on the changes in AS risk factors. A post-hoc test with Bonferroni correction was used to identify significant differences when a significant effect or interaction was identified.

We used machine learning algorithms to identify the optimal panels of lipid markers that could enable the identification of potential responders (i.e., participants who demonstrated favorable changes in AS risk factors within themselves after the intervention) from non-responders (i.e., participants who showed no decrease in AS risk factors following the intervention). The differences in lipid markers between the responders and non-responders were also assessed using the Wilcoxon test. Qualified data from plasma lipid species and clinical indices at baseline were analyzed separately. Metabolite features with *p* < 0.1 were selected for input into a random forest algorithm, integrated within a repeated double cross-validation framework with unbiased variable selection (using the R package “MUVR”), to identify optimal lipid panels predictive of a reduction in AS [32]. This approach has been validated to enhance predictive power and reduce false positives. Predictive accuracy was evaluated on the basis of the misclassification rate (%) and the area under the receiver operating characteristic curve (AROC, 95% CI).

This study revealed the secondary outcomes of a registered randomized placebo-controlled clinical trial (No. ChiCTR1800015383). The sample size calculations originally estimated that 12 participants were required in each group to provide a statistical power of 85% and detect an effect size of 0.26 [33] for differences in the improvement in insulin resistance after vitamin D supplementation. Sixty patients with diabetes were required to account for a 20% loss to follow-up. Sample size calculations were performed using G*Power software version 3.1.9.2 [34].

## 3. Results

### 3.1. Baseline Characteristics of the Participants

**Figure A1:**
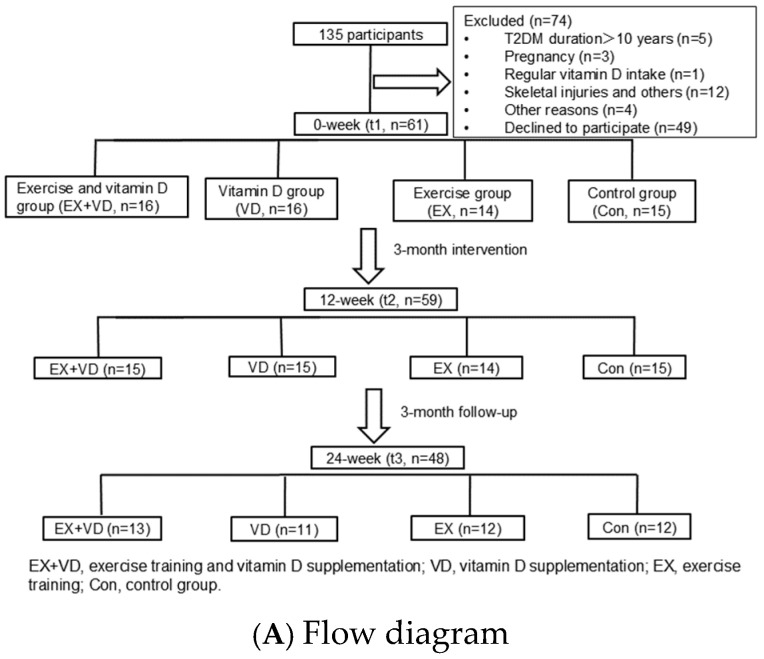

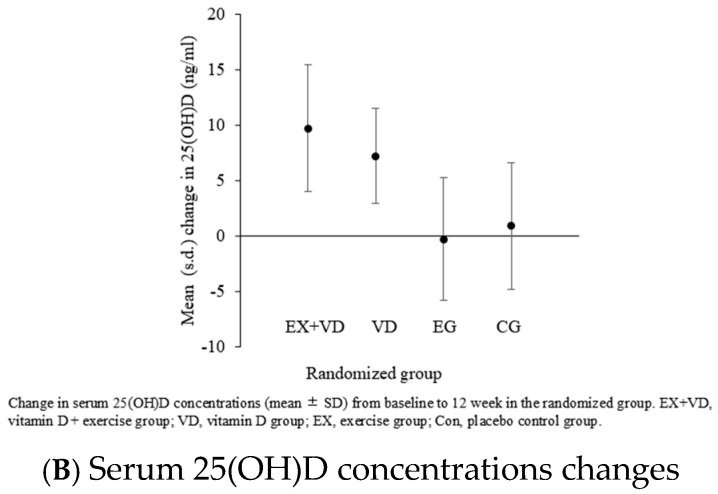
Flow diagram of participants (**A**) and changes in serum 25(OH)D concentrations during the intervention (**B**).

The baseline characteristics of the study participants are presented in Table 1. The overall average age was 50.1 ± 7.3 years, and the mean BMI was 25.9 ± 3.6 kg/m^2^ at baseline. Among the patients, 44 (71.2%) were males. Ultimately, 61 participants (EX + VD, *n* = 16; VD, *n* = 16; EX, *n* = 14; CON, *n* = 15) were included. There were no significant differences in age, sex, BMI, BF%, 25(OH)D concentrations, and AS indicators at baseline among the groups. During the intervention, one patient in VD and another in EX + VD withdrew from the study due to personal reasons; finally, 59 participants were included in the analysis. For more details, please see Appendix A, Figure A1A.

Among the 29 participants who completed the interventions in the EX + VD and EX groups, 20 participated in exercise two to three times per week, and 9 exercised less than two times per week. No significant difference in the frequency of exercise participation was observed among the groups. No intervention-related complaints were reported.

### 3.2. Effects of Exercise and Vitamin D on Serum 25(OH)D Concentrations and AS Indicators

Serum 25(OH)D concentrations increased from 17.7 ± 6.4 ng/mL to 27.5 ± 7.8 ng/mL in the EX + VD group and from 19.0 ± 7.7 ng/mL to 26.3 ± 7.7 ng/mL in the VD group, whereas no changes were observed in the EX and CON groups (Figure A1B). Vitamin D supplementation significantly slowed the reduction in ABI (mean ± SD: EX + VD and VD groups, −0.001 ± 0.058; EX + CON group, −0.047 ± −0.089; *p* = 0.03) (Table 2). This effect persisted even after adjusting for basal values and BF%. Although a significant effect of exercise on arm SBP was observed (*p* = 0.049), this association disappeared after adjusting for baseline SBP. No significant interactive effects of exercise or vitamin D were observed for any variables. Furthermore, we incorporated basal medication status as a covariate in the analysis, which did not have a statistically significant impact on the results.

### 3.3. Individual Responses to the Intervention on the Reduction in AS and Baseline Predictions

Because there might be high interindividual variability in these clinical indicators upon intervention, we evaluated each individual response. Here, 7, 11, 10 and 8 patients from EX group benefitted with regard to arm SBP, ankle SBP, ankle DBP, and baPWV, respectively, all of whom were considered responders. In the VD group, there were 10, 10, 9 and 7 responders, respectively. In the EX + VD group, there were six, six, eight, and seven responders, respectively (Figure 1).

Compared with traditional clinical indicators, the optimal metabolic predictive markers selected by the model were 100% better for arm SBP in the EX group (Figure 1A). The baseline levels of SM d44:6, LPE 18:2, and Hex2Cer 29:0 significantly increased the changes in arm SBP caused by EX (prediction rate = 71.43%, AROC = 0.672, 95% CI: 0.643–0.701), which was significantly better than the traditional clinical indicators (prediction rate, 35.71%; AROC, 0.632; 95% CI: 0.603–0.661). For patients with higher levels of SM d44:6, LPE 18:2, and Hex2Cer 29:0 at baseline, the exercise intervention facilitated improvements in arm SBP.

The optimal metabolic predictive markers and traditional clinical indicators selected by the model to predict the response of ankle SBP (lipids: prediction rate, 71.43%; AROC, 0.506; 95% CI: 0.469–0.544; clinical indices: prediction rate, 71.43%; AROC, 0.706; 95% CI: 0.675–0.737) or DBP (lipids: prediction rate, 78.57%; AROC, 0.684; 95% CI: 0.653–0.716; clinical indices: prediction rate, 71.43; AROC, 0.529; 95% CI: 0.496–0.562) in the EX group were similar (Figure 1B,C).

Compared with the traditional clinical indicators, the optimal metabolic predictive markers selected by the model improved the ability to predict the response to changes in baPWV in the VD group by 100% (Figure 1D). Cer 33:1 and GM3 44:4 significantly improved the change in baPWV caused by vitamin D supplementation (prediction rate, 76.92%; AROC, 0.750; 95% CI: 0.724–0.776), which was better than the traditional clinical indicators (prediction rate, 38.46%; AROC, 0.634; 95% CI: 0.604–0.664). In patients with lower baseline Cer 33:1 and GM3 44:4 levels, vitamin D supplementation improved the baPWV.

Moreover, the prediction performance was significantly improved when lipid predictors were added to the clinical indices for the four indicators above (i.e., arm SBP AUC, AROC = 0.951 [0.941–0.962]; ankle DBP AUC, AROC = 0.949 [0.939–0.959]; baPWV AROC = 0.9313 [0.917–0.945]) compared with using either lipid or clinical indices alone (Figure 1A–D).

## 4. Discussion

Although vitamin D supplementation failed to provide additional benefits on AS risk beyond exercise training, we found that 12 weeks of vitamin D supplementation could improve the ABI even after adjustment for basal ABI and BF%. Significantly, we observed high inter-individual variability in AS-related traits (i.e., arm SBP, ankle SBP, ankle DBP, and baPWV), which may explain the lack of any additional effects of exercise and/or vitamin D supplementation on AS risk to a great extent. We subsequently identified several lipid species that enhanced the prediction of reductions in AS compared with traditional lipid risk factors. Furthermore, the predictive performance substantially improved when lipid predictors were combined with clinical indices rather than relying solely on either lipids or clinical indices.

This research suggests that serum 25(OH)D concentrations increased by 45.6%, and vitamin D deficiency decreased from 65.6% at baseline to 20.0% after supplementation. This finding is similar to that of previous studies [35]. The increase in serum 25(OH)D had no effect on total BP but increased arm SBP; however, this effect disappeared after adjusting for basal arm SBP in the present study. These results are aligned with evidence from three recent meta-analyses [36,37,38].

The baPWV and ABI are useful predictors of arterial diseases in patients with diabetes [39,40]. Due to its broad distribution in vascular endothelial cells, vascular smooth muscle cells, and cardiomyocytes, the role of VDR in arterial diseases has received extensive attention [9]. Previous studies have reported that lower concentrations of 25(OH)D were associated with decreased ABI or increased baPWV in patients with peripheral arterial disease or diabetes [41,42,43,44,45,46]. In the present study, while the ABI changes remained within normal ranges, the inhibition of the reduction in ABI after vitamin D supplementation was statistically significant. The associations persisted even after adjusting for baseline ABI values and BF%, indicating that the effect was not due to random variation. Furthermore, a decrease in ABI values would suggest a reduction in the risk for arterial diseases.

Furthermore, we observed that the serum 25(OH)D concentrations were positively related to changes in the ABI (r = 0.259, *p* = 0.05). We did not observe any effect of vitamin D on baPWV, which is aligned with the evidence from two RCTs [13,47]. Furthermore, a recent meta-analysis of 10 RCTs systematically reported that vitamin D supplementation could only improve the PWV in studies with an intervention duration of over 4 months and a daily dose of vitamin D of over 2000 IU [48]. These results revealed that long-term interventions with higher doses of vitamin D supplementation might have a greater preventive influence on AS.

Numerous studies have indicated that exercise plays a crucial role in the prevention of AS due to improvements in vasoactive substances and endothelial function; however, these effects seem to be more obvious with combined endurance and resistance exercise or high-intensity exercise interventions [49,50,51,52,53]. Recently, a systematic review reported that high-intensity exercise interventions could improve AS, as measured by the PWV; however, it was not observed in the moderate-intensity exercise intervention group [53]. This was also supported by a recent 12-week RCT conducted on patients with diabetes [49]. Moreover, it was found that endurance exercise or resistance exercise can increase VDR levels, which may further augment the effects of vitamin D supplementation.

In addition to the effects of exercise and vitamin D on the regulation of AS indicators, we found significant differences in the changes in AS indicators among individuals. These results confirmed the necessity and importance of individualized combined nutrition and exercise therapy programs. Compared with traditional clinical indicators, baseline lipid prediction can significantly improve the predictive ability for changes in AS indicators. Importantly, the prediction performance significantly improved when lipid predictors were included alongside clinical indices, compared with using lipid or clinical indices independently.

We also observed significant variability in individual responsiveness to changes in AS risk factors among participants undergoing the interventions. In the VD + EX group, only about half of the participants responded positively in terms of arm SBP, ankle SBP, ankle DBP, and baPWV. These findings align with and extend existing evidence on the inter-individual differences in response to diet or exercise interventions, underscoring the necessity and importance of personalized exercise programs [54,55]. On the other hand, the potential effect of this variability on the statistical power is also crucial to be noted.

SMs are prevalent sphingolipids in mammalian cells and are pivotal for regulating the plasma membrane and cellular cholesterol homeostasis. Numerous studies have explored the relationship between circulating plasma SM levels and cardiovascular disease (CVD) risk, yet the results have been inconsistent. Fernandez et al. identified SM (38:2) as the only lipid species in this class linked to a higher risk of developing CVDs [56]. Conversely, another study reported that individuals with impaired fasting glucose or Type 2 diabetes had significantly lower serum concentrations of glycerophospholipids and SMs compared with healthy controls, even after adjusting for age, sex, and BMI [57]. The authors of this study proposed that a decrease in the SM pool in diabetes might lead to increased oxidative stress and decreased insulin secretion, resulting in hyperglycemia. Our findings also support this hypothesis. We observed that higher SM (44:6) levels at baseline could substantially improve the prediction rate in the EX group. Similarly, prior evidence suggests that lower levels of LPEs and Cers are associated with a higher risk of CVDs. Pickens et al. found that several classes of LPLs (i.e., LPC, LPE, and LPS) had an inverse relationship with BMI, waist circumference, leptin, and C-peptide [58]. Plasma ceramide (14:0) is regarded as the strongest predictor of insulin resistance in obesity and diabetes because its decreased level is negatively correlated with increased insulin sensitivity [59]. A 12-week aerobic and resistance exercise training program significantly increased the levels of diacylglycerol (32:2) and ceramide (d18:1/24:0) and the total PC and PE contents in an RCT involving 45 obese women [60].

For baPWV, lower levels of two selected lipids, Cer (33:1) and GM3 (44:4), at baseline were found to enhance the predictive power by 100% in the VD group compared with the clinical indices. This finding is consistent with previous evidence. A prior study has indicated that vitamin D supplementation leads to alterations in plasma levels of C18 chain length-specific dihydroceramides (dhCer) and ceramides (Cer) in patients with diabetes [61]. A follow-up study of 12.9 years among 2627 ethnically Chinese Singaporeans revealed that higher circulating total monohexosylceramides, total long-chain sphingolipids, and total 18:1 sphingolipids were associated with a higher CVD risk [62]. Vitamin D supplementation has been shown to decrease plasma triglyceride levels [63]. A RCT showed that 600, 2000, and 4000 IU/day of vitamin D supplementation increased the serum levels of C18Cer and C18SM in overweight/obese African Americans [48]. Furthermore, GM3 is a subgroup of gangliosides that plays a pivotal role in cell signaling, inflammation, and insulin resistance. Gangliosides are known for their ability to downregulate both acute and chronic inflammatory signals. In human serum, GM3 (d18:1/h24:1) is highly correlated with the development of diabetes [64].

The strengths of the present trial included its 2 × 2 factorial RCT design, high participant retention, and high adherence to the intervention. Moreover, our study is the first to investigate the effect of vitamin D alone and in combination with exercise on the reduction in AS in patients with diabetes and to explore the underlying mechanism from the perspective of lipidomics. The present study has several limitations, including its relatively short duration, which may have restricted our capacity to identify significant changes in lipid species. Nonetheless, previous research has shown that alterations in blood metabolic profiles can be observed within 12 weeks [65]. Second, although the participants were ordered not to change their diet and medication regimens during the interventions, the potential impact of such changes could not be omitted. Third, the change in ABI among our subjects was not a significant result, although the observed numerical reduction may still suggest a potential reduction in the risk to some extent. The main reason can mainly be attributed to the fact that our subjects were those with mild or early-stage diabetes. Fourth, the outcome reported in this study was a secondary result of a registered RCT. Lastly, while the sample size was relatively small, a post hoc power calculation indicated at least a 90% probability of detecting the interactive effect on blood pressure, defined as statistically significant at *p* < 0.05 at the endpoints. Further research with well-designed RCTs that involve larger sample sizes and longer intervention durations is needed to focus on risk factors of arterial stiffness.

## 5. Conclusions

In summary, we found that 12 weeks of vitamin D supplementation may have beneficial effects on AS. However, vitamin D supplementation failed to provide additional benefits beyond exercise training, which may be attributed to the high inter-individual variability in AS-related traits. Moreover, several lipid species could enhance the predictive ability for the reduction in arterial stiffness compared with traditional lipid risk factors.

## Figures and Tables

**Table 1:** Subjects’ characteristics at baseline.

Variables	Overall	EX + VD	VD	EX	Con	*p*
	*n* = 59	*n* = 15	*n* = 15	*n* = 14	*n* = 15	
Age (years)	50.1 ± 7.3	50.6 ± 6.6	51.2 ± 7.2	47.9 ± 8.6	50.6 ± 7.0	0.620
Male (%)	44 (71.2%)	10 (60.0%)	12 (73.3%)	10 (71.4%)	12 (80.0%)	0.711
25(OH)D (ng/mL)	17.4 ± 6.2	17.7 ± 6.4	19.0 ± 7.7	16.9 ± 6.1	15.9 ± 4.4	0.572
Height (cm)	167.4 ± 7.6	165.1 ± 8.1	167.6 ± 8.5	169.1 ± 7.7	167.8 ± 6.1	0.550
Weight (kg)	72.9 ± 13.1	68.9 ± 13.1	71.4 ± 13.1	74.7 ± 15.1	76.9 ± 10.9	0.364
BMI (kg/m^2^)	25.9 ± 3.6	25.1 ± 3.0	25.3 ± 3.3	26.1 ± 4.8	27.2 ± 3.2	0.363
Body fat (%) ^a^	30.3 ± 7.7	32.0 ± 6.1	27.9 ± 9.2	30.9 ± 8.6	30.3 ± 6.6	0.531
Body muscle (g)	47,651 ± 9036	44,167 ± 9380	48,307 ± 9540	47,915 ± 8352	50,418 ± 8522	0.419
Physical activity (MET-min/week)	3578 ± 3454	3164 ± 2413	4065 ± 4583	4334 ± 4286	2801 ± 1976	0.596
Sun exposure weekly score	14.8 ± 7.3	16.5 ± 8.3	13.6 ± 4.1	16.1 ± 8.8	13.2 ± 7.3	0.506
Diabetes duration (years)	3.5 ± 2.4	3.3 ± 2.4	3.9 ± 2.4	3.1 ± 2.5	3.7 ± 2.6	0.856
HOMA-IR	4.0 ± 2.6	3.9 ± 3.0	3.4 ± 2.6	3.9 ± 2.3	4.9 ± 2.6	0.587
SBP_arm (mmHg)	128.3 ± 16.9	118.6 ± 11.4	132.1 ± 16.5	131.5 ± 17.6	131.4 ± 18.9	0.080
DBP_arm (mmHg)	79.3 ± 9.9	74.7 ± 8.8	81.5 ± 9.3	80.1 ± 11.4	80.9 ± 9.7	0.220
SBP_ankle (mmHg)	148.4 ± 21.7	136.7 ± 18.0	152.5 ± 23.0	152.0 ± 23.4	152.6 ± 20.0	0.116
DBP_ankle (mmHg)	78.2 ± 10.3	74.3 ± 9.4	78.2 ± 11.0	80.0 ± 12.2	80.4 ± 8.0	0.354
baPWV (cm/s) ^a^	1482 ± 218	1423 ± 226	1505 ± 209	1456 ± 206	1545 ± 232	0.458
ABI	1.14 ± 0.06	1.13 ± 0.07	1.14 ± 0.06	1.14 ± 0.06	1.15 ± 0.06	0.882

Data are means ± standard deviation (SD) or *n* (%). EX + VD, vitamin D supplementation and exercise training; VD, vitamin D supplementation; EX, exercise training; CON, control group; HbA1c, glycated hemoglobin; MET, metabolic equivalent; HOMA-IR, homeostasis model assessment of insulin resistance. $\mathrm{HOMA}-\mathrm{IR}=\frac{\mathrm{Fasting}\;\mathrm{Insulin}\;\left(\mu U/\mathrm{mL}\right)\times \;\mathrm{Fasing}\;\mathrm{Glucose}\;\left(\mathrm{mg}/\mathrm{dL}\right)}{405}$. The differences among the groups were compared using one-way analysis of variance for continuous variables and chi-squared tests for categorical variables. The paired Student’s *t*-test was used to compare AS indicators within groups. ^a^, only 14 samples in CON were included.

**Table 2:** Mean differences (95% CI) of changes in arterial stiffness factors from the baseline with each group using 2 × 2 factorial ANOVA.

Variables	EX + VD	VD	EX	Con	*p*		
	*n* = 15	*n* = 15	*n* = 14	*n* = 15	*p*_vitamin D_	*p*_exercise_	*p*_interaction_
Δ Weight (kg) ^a^	−0.3 (−1.3, 0.7)	−0.3 (−1.6, 1.0)	−1.1 (−1.9, −0.2)	−0.2 (−1.1, 0.7)	0.516	0.386	0.341
Δ BMI (kg/m^2^) ^a^	0.7 (−1.0, 2.5)	0.1 (−0.6, 0.4)	−0.3 (−0.7, 0.0)	−0.1 (−0.4, 0.3)	0.245	0.522	0.211
Δ 25(OH)D (ng/mL)	9.7 (6.6, 12.9)	7.3 (4.7, 9.9)	−0.3 (−3.5, 2.8)	0.9 (−2.3, 4.0)	**<0.001**	0.641	0.185
Δ Body fat (%)	−1.0 (−2.1, 0.2)	0.3 (−1.0, 1.7)	−1.1 (−2.0, −0.2)	−0.4 (−1.1, 0.4)	0.262	**0.032**	0.428
Δ Body fat (g)	−739 (−1614, 137)	127 (−1100, 1355)	−1027 (−1775, −279)	−295 (−862, 271)	0.301	**0.049**	0.776
Δ Body muscle (g)	529 (−194, 1252)	10 (−640, 661)	414 (−404, 1232)	113 (−485, 711)	0.758	0.134	0.505
Δ HOMA-IR	−0.9 (−2.4, 0.5)	−0.2 (−1.3, 0.8)	−0.2 (−1.6, 1.3)	0.8 (−1.5, 3.0)	0.446	0.274	0.751
Δ SBP_arm (mmHg) ^a^	5.7 (−0.9, 12.2)	−6.8 (−15.1, 1.5)	−1.2 (−7.1, 4.6)	−1.8 (−8.8, 5.2)	0.775	**0.049**	0.072
Δ DBP_arm (mmHg) ^a^	2.2 (−3.9, 8.3)	−4.2 (−8.2, −0.3)	−0.8 (−6.2, 4.7)	−2.7 (−5.5, 0.1)	0.751	0.064	0.312
Δ SBP_ankle (mmHg) ^a^	4.8 (−3.2, 12.8)	−8.5 (−18.1, 1.1)	−6.7 (−13.2, −0.1)	−7.2 (−16.1, 1.6)	0.108	0.055	0.055
Δ DBP_ankle (mmHg) ^a^	1.1 (−4.0, 6.3)	−3.1 (−8.0, 1.9)	−3.4 (−7.7, 0.8)	−1.5 (−4.5, 1.5)	0.467	0.580	0.139
Δ baPWV(cm/s) ^b^	11.7 (−74.1, 97.4)	20.0 (−92.3, 132.2)	−47.1 (−118.0, 23.7)	−44.3 (−119.8, 31.2)	0.131	0.891	0.946
Δ ABI	0.00 (−0.04, 0.03)	0.00 (−0.03, 0.04)	−0.05 (−0.10, 0.00)	−0.04 (−0.09, 0.00)	**0.026**	0.763	0.969

Data are the mean differences (95% confidence interval). A 2 × 2 factorial design was employed to assess the impact of vitamin D supplementation and an exercise intervention on changes in AS indicators. When a significant effect or interaction was observed, a post hoc test with Bonferroni correction was applied to determine significant differences. *p*_vitamin D_, the main effect of vitamin D; *p*_exercise_, the main effect of exercise; *p*_interaction_, the interactive effect of vitamin D combined with exercise. Δ, changes from the endpoint to the pre; EX + VD, vitamin D supplementation and exercise training; VD, vitamin D supplementation; EX, exercise training; CON, control group; BMI, body mass index; HbA1c, glycated hemoglobin; HOMA-IR, homeostasis model assessment of insulin resistance; AUC, area under the curve during the oral glucose tolerance test; SBP, systolic blood pressure; DBP, diastolic blood pressure; baPWV, brachial–ankle pulse wave velocity; ABI, ankle–branchial index; CI, confidence interval. ^a^, *n* = 14 in VD; ^b^, *n* = 13 in VD and *n* = 14 in CON were included.

## Data Availability

The data used in this study are not publicly available due to ethical reasons; the corresponding author can provide further information on these data upon reasonable request.

## Abbreviations

ABI	Ankle–brachial index
AROC	Area under the receiver operating characteristic curve
AS	Arterial stiffness
baPWV	Brachial–ankle pulse wave velocity
BF%	Body fat percentage
BMI	Body mass index
CI	Confidence interval
CON	Control group
CVD	Cardiovascular disease
DBP	Diastolic blood pressure
EX	Exercise group
HRmax	Maximal heart rate
PWV	Pulse wave velocity
SBP	Systolic blood pressure
SM	Sphingomyelin
T2DM	Type 2 diabetes melitum
VD	Vitamin D group
LPE	Lipoprotein
Hex2Cex	Hexosyl (2) ceramide

## Appendix A

**Table A1:** The protocol of endurance exercise training.

Stage	HRmax (%)	RPE	Time
Week 1	65–70%	11–13	60 min (including 5 min warm-up and 5 min of recovery exercise)
Week 2–4	70–75%	11–13	60 min (including 5 min warm-up and 5 min of recovery exercise)
Week 5–8	75–80%	12–13	60 min (including 5 min warm-up and 5 min of recovery exercise)
Week 9–12	80%	13	60 min (including 5 min warm-up and 5 min of recovery exercise)

Note: HRmax, maximum heart rate; RPE, rating of perceived exertion.
